# The Exchange of Serum.—The H. K. Mulford Company State the Conditions Making Exchange Necessary

**Published:** 1905-11

**Authors:** 

**Affiliations:** Milton Campbell, Prest., Philadelphia


### CORRESPONDENCE.

### The Exchange of Serum.—The H. K. Mulford Company State the Conditions Making Exchange Necessary.

*Editor Buffalo Medical Journal:*

SIR—Our attention has been called to an editorial appearing in "American Medicine" of September 9, 1905, criticising the policy of exchanging unused and out-of-date serum for a fresh supply, comparing this custom with that of foreign serums where this privilege is not extended. Every producer of antitoxin would be glad to have this exchange system done away with were it possible to do so without grave injury to the medical profession.

The exchange privilege means a constant and large loss to the manufacturers, but speaking for ourselves with the experience of a large number of years of producing and handling antitoxins, in our judgment the exchange privilege must be extended for the following reasons:

First.—Epidemics of Diphtheria are very uncertain and in consequence the demand for antitoxin is uncertain. Physicians therefore cannot afford to purchase antitoxin and take the risk of having occasion to use it before it gets out of date and should it not be used be an absolute loss to them on account of not being exchangeable for fresh stock.

Second.—The same argument applies to the druggist who under the present arrangements being protected against loss is willing and does carry a sufficient supply to meet these emergency conditions. Were antitoxin not exchangeable it would result in a tendency on the part of the manufacturer to give serums an extra long dating and the antitoxin in consequence would lose a portion, or in many instances a large part, of its antitoxic properties, and the value of the product would become uncertain and in time the product itself would become discredited. Also there would be a strong temptation for the drug trade to supply out-dated serum and on the part of the physician to use this serum, if in his office, with the knowledge that it could not be exchanged for fresh serum.

Third.—From the above reasons it is plainly seen that neither the physician or the druggist could afford or would be willing to stock antitoxins with a loss staring them in the face under the conditions of no exchange. In consequence, as the remedy is an emergency one and prompt employment is neccessary to get the best results, there would be a number of lives sacrificed through the fact that antitoxin was not obtainable in these cases of emergency. The medical profession would be poorly served and we are confident they would not accept a policy which would lead to such unfortunate conditions.

The handling of antitoxin abroad differs very largely from that in our own country, and their customs are different. What is frequently done in Europe and is satisfactory would not be so in our own country. Europeans have tried for years to introduce their serums in this country, and have largely failed through the fact that their products were not exchangeable for fresh stock. The American physicians have received more liberal treatment and absolute protection from the manufacturers of antitoxin in the past than in any other country, and we do not believe they would be satisfied with any system which would curtail their privileges which they have enjoyed in the past, and the protection which it not only affords them but their patients, nor do we believe that the manufacturer would look upon it as good policy for the sake of increasing his profit to adopt this course, by which the exchange privilege would not be extended.

Where the question of life or death is at stake, as in the use of such an important remedy as antitoxin, its freshness has always been recognised as one of the prime requisites for its activity. The medical profession has always justly discriminated between antitoxins and have been guided more by the excellence of the product and its freshness and reliability than the price. So much depends upon its prompt action that it would make any policy of long dated serum, or serum which is not exchangeable for a fresh supply, unpopular, hence we are glad to place these facts before you.

H. K. MULFORD COMPANY,
Milton Campbell, Prest.

Philadelphia, September 26, 1905.

---

PATHOLOGY AND THERAPY OF DIABETES MELLITUS.—Prof. Carl von Noorden, of Frankfort-on-the-Main, writing on this subject (*Deutsche Aerzte-Zeitung*, November 15, 1902), says: The last point which I wish to discuss is the question of carbohydrate nutriment for diabetics. That various carbohydrates act very differently on the glycosuria, has long been known and has especially been subject to study by Kuelz. In practice, however, it is only important that Levulose (Schering's) is a great deal better assimilated by diabetics than any other carbohydrate in our foodstuffs

